# Prevalence, Distribution, and Impact of Mild Cognitive Impairment in Latin America, China, and India: A 10/66 Population-Based Study

**DOI:** 10.1371/journal.pmed.1001170

**Published:** 2012-02-07

**Authors:** Ana Luisa Sosa, Emiliano Albanese, Blossom C. M. Stephan, Michael Dewey, Daisy Acosta, Cleusa P. Ferri, Mariella Guerra, Yueqin Huang, K. S. Jacob, Ivonne Z. Jiménez-Velázquez, Juan J. Llibre Rodriguez, Aquiles Salas, Joseph Williams, Isaac Acosta, Maribella González-Viruet, Milagros A. Guerra Hernandez, Li Shuran, Martin J. Prince, Robert Stewart

**Affiliations:** 1 National Institute of Neurology and Neurosurgery, Autonomous National University of Mexico, Mexico City, Mexico; 2 Laboratory of Epidemiology, Demography and Biometry, National Institute on Aging, Bethesda, Maryland, United States of America; 3 University of Cambridge, Cambridge, United Kingdom; 4 King's College London (Institute of Psychiatry), London, United Kingdom; 5 Universidad Nacional Pedro Henriquez Ureña (UNPHU), Internal Medicine Department, Geriatric Section, Santo Domingo, Dominican Republic; 6 Universidad Peruana Cayetano Heredia, Instituto de la Memoria y Desordenes Relacionados, Peru; 7 Peking University, Institute of Mental Health, Beijing, China; 8 Christian Medical College, Vellore, India; 9 UPR, School of Medicine, San Juan, Puerto Rico; 10 Medical University of Havana, Cuba; 11 Medicine Department, Caracas University Hospital, Faculty of Medicine, Universidad Central de Venezuela, Caracas, Venezuela; 12 Institute of Community Health, Voluntary Health Services, Chennai, India; 13 Psy D Program Carlos Albizu University, San Juan, Puerto Rico; 14 Policlinico Universitario 27 de Noviembre, Havana, Cuba; Mount Sinai School of Medicine, United States of America

## Abstract

**Background:**

Rapid demographic ageing is a growing public health issue in many low- and middle-income countries (LAMICs). Mild cognitive impairment (MCI) is a construct frequently used to define groups of people who may be at risk of developing dementia, crucial for targeting preventative interventions. However, little is known about the prevalence or impact of MCI in LAMIC settings.

**Methods and Findings:**

Data were analysed from cross-sectional surveys established by the 10/66 Dementia Research Group and carried out in Cuba, Dominican Republic, Peru, Mexico, Venezuela, Puerto Rico, China, and India on 15,376 individuals aged 65+ without dementia. Standardised assessments of mental and physical health, and cognitive function were carried out including informant interviews. An algorithm was developed to define Mayo Clinic amnestic MCI (aMCI). Disability (12-item World Health Organization disability assessment schedule [WHODAS]) and informant-reported neuropsychiatric symptoms (neuropsychiatric inventory [NPI-Q]) were measured. After adjustment, aMCI was associated with disability, anxiety, apathy, and irritability (but not depression); between-country heterogeneity in these associations was only significant for disability. The crude prevalence of aMCI ranged from 0.8% in China to 4.3% in India. Country differences changed little (range 0.6%–4.6%) after standardization for age, gender, and education level. In pooled estimates, aMCI was modestly associated with male gender and fewer assets but was not associated with age or education. There was no significant between-country variation in these demographic associations.

**Conclusions:**

An algorithm-derived diagnosis of aMCI showed few sociodemographic associations but was consistently associated with higher disability and neuropsychiatric symptoms in addition to showing substantial variation in prevalence across LAMIC populations. Longitudinal data are needed to confirm findings—in particular, to investigate the predictive validity of aMCI in these settings and risk/protective factors for progression to dementia; however, the large number affected has important implications in these rapidly ageing settings.

*Please see later in the article for the Editors' Summary*

## Abstract 2

A set of cross-sectional surveys carried out in Cuba, Dominican Republic, Peru, Mexico, Venezuela, Puerto Rico, China, and India reveal the prevalence and between-country variation in mild cognitive impairment at a population level.

## Editors' Summary

**Background:**

Currently, more than 35 million people worldwide have dementia, a group of brain disorders characterized by an irreversible decline in memory, problem solving, communication, and other “cognitive” functions. Dementia, the commonest form of which is Alzheimer's disease, mainly affects older people and, because more people than ever are living to a ripe old age, experts estimate that, by 2050, more than 115 million people will have dementia. At present, there is no cure for dementia although drugs can be used to manage some of the symptoms. Risk factors for dementia include physical inactivity, infrequent participation in mentally or socially stimulating activities, and common vascular risk factors such as high blood pressure, diabetes, and smoking. In addition, some studies have reported that mild cognitive impairment (MCI) is associated with an increased risk of dementia. MCI can be seen as an intermediate state between normal cognitive aging (becoming increasingly forgetful) and dementia although many people with MCI never develop dementia, and some types of MCI can be static or self-limiting. Individuals with MCI have cognitive problems that are more severe than those normally seen in people of a similar age but they have no other symptoms of dementia and are able to look after themselves. The best studied form of MCI—amnestic MCI (aMCI)—is characterized by memory problems such as misplacing things and forgetting appointments.

**Why Was This Study Done?:**

Much of the expected increase in dementia will occur in low and middle income countries (LAMICs) because these countries have rapidly aging populations. Given that aMCI is frequently used to define groups of people who may be at risk of developing dementia, it would be useful to know what proportion of community-dwelling older adults in LAMICs have aMCI (the prevalence of aMCI). Such information might help governments plan their future health care and social support needs. In this cross-sectional, population-based study, the researchers estimate the prevalence of aMCI in eight LAMICs using data collected by the 10/66 Dementia Research Group. They also investigate the association of aMCI with sociodemographic factors (for example, age, gender, and education), disability, and neuropsychiatric symptoms such as anxiety, apathy, irritability, and depression. A cross-sectional study collects data on a population at a single time point; the 10/66 Dementia Research Group is building an evidence base to inform the development and implementation of policies for improving the health and social welfare of older people in LAMICs, particularly people with dementia.

**What Did the Researchers Do and Find?:**

In cross-sectional surveys carried out in six Latin American LAMICS, China, and India, more than 15,000 elderly individuals without dementia completed standardized assessments of their mental and physical health and their cognitive function. Interviews with relatives and carers provided further details about the participant's cognitive decline and about neuropsychiatric symptoms. The researchers developed an algorithm (set of formulae) that used the data collected in these surveys to diagnose aMCI in the study participants. Finally, they used statistical methods to analyze the prevalence, distribution, and impact of aMCI in the eight LAMICs. The researchers report that aMCI was associated with disability, anxiety, apathy, and irritability but not with depression and that the prevalence of aMCI ranged from 0.8% in China to 4.3% in India. Other analyses show that, considered across all eight countries, aMCI was modestly associated with being male (men had a slightly higher prevalence of aMCI than women) and with having fewer assets but was not associated with age or education.

**What Do These Findings Mean?:**

These findings suggest that aMCI, as diagnosed using the algorithm developed by the researchers, is consistently associated with higher disability and with neuropsychiatric symptoms in the LAMICs studied but not with most sociodemographic factors. Because prevalidated and standardized measurements were applied consistently in all the countries and a common algorithm was used to define aMCI, these findings also suggest that the prevalence of aMCI varies markedly among LAMIC populations and is similar to or slightly lower than the prevalence most often reported for European and North American populations. Although longitudinal studies are now needed to investigate the extent to which aMCI can be used as risk marker for further cognitive decline and dementia in these settings, the large absolute numbers of older people with aMCI in LAMICs revealed here potentially has important implications for health care and social service planning in these rapidly aging and populous regions of the world.

**Additional Information:**

Please access these Web sites via the online version of this summary at http://dx.doi.org/10.1371/journal.pmed.1001170.

- Alzheimer's Disease International is the international federation of Alzheimer associations around the world; it provides links to individual associations, information about dementia, and links to three World Alzheimer Reports; information about the 10/66 Dementia Research Group is also available on this web site
- The Alzheimer's Society provides information for patients and carers about dementia, including information on MCI and personal stories about living with dementia
- The Alzheimer's Association also provides information for patients and carers about dementia and about MCI, and personal stories about dementia
- A BBC radio program that includes an interview with a man with MCI is available
- MedlinePlus provides links to further resources about MCI and dementia (in English and Spanish)

## Introduction

Ageing [1] and the health transition in low- and middle-income countries (LAMICs) are responsible for an unprecedented increase in the prevalence and societal impact of noncommunicable diseases, including dementia [2]. Large numbers of people with dementia currently live in LAMICs [3],[4] with prevalence estimates comparable to those of the Western world [5]. At present, disease-modifying drugs are not available [6] and symptomatic medications have been found to have only modest benefit [7]. Primary prevention of dementia is therefore of great importance [8].

Mild cognitive impairment (MCI) is an intermediate state between normal cognitive ageing and dementia [9]. Identification of MCI is thought to be crucial to early intervention. Indeed, in some studies MCI is associated with an increased risk of dementia [10], as well as with future disability [11] and mortality [12]. Such associations, however, do vary according to the nature of the sample (clinical versus population-based), the case definition of MCI applied, the assessment procedures used for operationalizing component criteria [13]–[15], and, potentially, the cultural background of participants [16],[17]. A recent review also suggested that MCI is associated with neuropsychiatric symptoms, cited as being of potential importance for defining subgroups at higher risk of developing dementia in the future [18].

In community-dwelling older adults the prevalence of amnestic MCI (aMCI), defined according to Petersen's revised criteria [10], ranges between 2.1% [19] and 11.5% [20] and is most commonly found to be around 3%–5% [21]–[33] with few exceptions in older samples [20],[34]–[36]. Reports of the community prevalence of aMCI have been predominantly derived from European and North American populations. To our knowledge, very few population-based studies have been published from LAMICs and those from Asia are controversial. Specifically, estimates of aMCI prevalence were similar to those found in Western countries in Kolkata, India (6%) [37] and in Chongqing, China (4.5%) [29], but higher prevalences were reported by Lee and colleagues in Malaysia (15.4%) [38] and by Kim et al. in South Korea (9.7%) [39].

Estimating the population prevalence of MCI in LAMICs is a public health priority as rapid demographic ageing is predicted to result in a large majority of people residing in these regions being at risk of dementia and cognitive decline. If so, this will have significant implications with regard to social support and future health care costs, especially as systems are not in place to cope with increased neurodegenerative disease and health resources at present are already extremely limited.

In this study, using data from the cross-sectional phase of the 10/66 Dementia Research Group (DRG) programme on dementia, noncommunicable diseases and ageing in LAMICs [40], we operationalized the Mayo Clinic–defined aMCI [10] construct and then estimated the prevalence of this condition in eight LAMICs, in addition to its sociodemographic correlates and associations with disability and neuropsychiatric symptoms.

## Methods

### Ethics Statement

Written informed consent, or witnessed oral consent in case of illiteracy, or next of kin written agreement in case of incapacity, was obtained from all participants. The appropriate Research Ethics Committees at King's College London and at all local countries approved the study protocol and the consent procedures.

### Sample

The 10/66 study has been described previously [40]. In brief, the study consisted of a series of cross-sectional one-phase geographic catchment area surveys, carried out in eight urban and rural sites in Peru, Mexico, China, and India, and in three urban sites in Cuba, the Dominican Republic, and Venezuela, between January 2003 and November 2007. The target sample size was 2,000 participants per country, in order to allow estimation of a typical dementia prevalence of 4.5% (SE 0.9%) with 80% power. All community-resident individuals aged 65+ y were eligible for inclusion. Using a process of full household enumeration, all residents aged 65+ y within catchment areas were approached by means of door-knocking and a reliable informant was required for inclusion. Being younger than 65 y was the only exclusion criteria, and weighted sampling procedures were not applied.

### Measurements

All participants completed the 10/66 standardized assessment at their place of residence. This consisted of participant and informant interviews and a physical examination, described in full elsewhere in an open-access publication [40]. Participant interviews included questionnaire measures of sociodemographic status, education and childhood environment, social networks and support, self-report measures of common physical disorders, health service use, and lifestyles (smoking, alcohol intake, diet, exercise), in addition to a fully structured diagnostic interview for mental disorder (Geriatric Mental State [GMS], described below). Physical examinations included measures of resting blood pressure, anthropometric measures, and a structured neurological examination. A battery of cognitive assessments was administered (described below) and an informant interview included structured questionnaires on cognitive decline and neuropsychiatric symptoms (both described below), as well as questions on care arrangements, caregiver strain and distress, financial implications of caregiving, and support received. The 10/66 study protocol was translated into Spanish, Tamil, and Mandarin, and minor adaptations were made by local clinicians fluent in English. Validation statistics for the assessments and procedures have been published [41]. The protocol included the GMS Examination [42],[43], an informant interview on all participants, a neurological examination, and a neuropsychological battery that comprised the following:

(1) The participant interview section of the Community Screening Instrument for Dementia (CSI “D”) [44]. This was developed as a screening instrument for dementia for use in cross-cultural settings in combination with the informant interview. The cognitive assessment covers multiple domains, including orientation to time and place, language, memory, praxis, and abstract thinking. It deliberately excludes literacy-dependent items. A memory subscale was derived from the CSI “D” using the items addressing immediate and delayed recall of a three word list, recall of the name of the interviewer, and recall of five elements of a short story (logical memory). (2) The Modified Consortium to Establish a Registry for Alzheimer's Disease (CERAD) ten-word-list learning task [45]. Six words: butter, arm, letter, queen, ticket, and grass were taken from the original CERAD battery English language list. Pole, shore, cabin, and engine were replaced with corner, stone, book, and stick, which were deemed more culturally appropriate for all sites in the 10/66 pilot phase (a wider sample that included the survey sites). In the learning phase, the list is read to the participant. Next, the participant is asked to immediately recall the words that they remember. This process is repeated three times, giving an immediate word list memory score, with a maximum total of 30. After a 5-min delay, the participant is again asked to recall the ten words with encouragement but no cues, giving a word list delayed recall score with a maximum total score of 10.

Demographic correlates analyzed against aMCI were age, gender, education, and number of assets. Participants' gender and stated age were recorded. Age was confirmed by the interviewer from official documentation and informant report, and any discrepancies resolved through further questions and clarification and, ultimately, by consensus within the research team. Illiteracy (inability to read and/or write), level of education (none/did not complete primary/completed primary/secondary/tertiary), and number of household assets (car, television, refrigerator, telephone, plumbed toilet, water, and electricity mains) were also recorded.

The impact of aMCI was quantified through investigating associations with disability and neuropsychiatric symptoms. Participant interviews included the 12-item WHO disability assessment schedule (WHODAS-12) [46], which assesses five activity-limitation domains (communication, physical mobility, self-care, interpersonal interaction, life activities and social participation). Two questions with scores ranging from 0 (no difficulty) to 4 (extreme difficulty) cover each domain, and the global standardized score ranges from 0 (not disabled) to 100 (maximum disability). Details on the WHODAS 2.0 validity and psychometric properties can be found elsewhere [47],[48]. The informant interview, as well as administering structured CSI “D” questions (regarding decline in memory or intelligence, activities of daily living, social and occupational functioning used for dementia diagnoses—summarized below and applied as an exclusion criteria), also included the neuropsychiatric inventory (NPI-Q) [49], and the following binary symptom categories were selected for analyses of associations with aMCI: depression, anxiety, apathy, irritability.

For analyses of associations of aMCI with disability and neuropsychiatric symptoms, the following covariates available in the dataset were used for adjusted models in addition to the four sociodemographic variables described above: depression (GMS), self-reported limiting physical impairments (arthritis, visual difficulties, hearing difficulties, respiratory disorders, heart problems, gastrointestinal problems, fainting episodes, limb paralysis, skin disorders), self-reported hypertension, self-reported stroke, psychotic disorder (GMS), self-reported regular pain.

### Case Definition of aMCI

Mayo Clinic–defined aMCI was diagnosed on the basis of the following criteria: (1) objective memory impairment beyond that expected for age; (2) subjective memory complaint; (3) no, or only mild impairment in core activities of daily living, and (4) no dementia. Each criterion was operationalized as follows.

#### Objective memory impairment

A composite memory score was created using results from the memory subscale of the CSI “D” [44], immediate and delayed word recall scores from the modified CERAD ten-word list [50]. For all tasks impaired performance was defined as a score 1.5 standard deviation (SD) or more below the mean adjusted for age and education. The 1.5-SD definition stems from that applied to define “abnormal memory performance” by Peterson et al. in 1999 [9], and has been recently recommended also by a National Institute on Aging-Alzheimer's Association workgroup [51]. Operationalization of MCI in other population-based studies has consistently followed this definition [25],[33],[52],[53], which has also been used to define other constructs such as “Cognitive Impairment No Dementia” [54]. The CERAD word list has been used in previous research [25]. Although, there have been controversies surrounding the MCI entity itself [55]–[58], they have not to our knowledge focused on the 1.5-SD threshold. Norms were derived from controls without dementia from the 24-centre 10/66 pilot study, which had found minimal geographic variation [41]. Participants were excluded if hearing impairment had prevented cognitive assessment.

#### Subjective memory impairment

An ordinal scale ranging from 0 to 6 was created by summing item scores from relevant questions in the GMS including: (1) Have you had any difficulty with your memory (0, no; 1, yes)? (2) Have you tended to forget names of your family or close friends/where you have put things (for each question: 0, no/transient; 1, noticed most days per week; 2, noticed daily)? (3) Do you have to make more efforts to remember things than you used to (0, no; 1, yes)? Using this scale, subjective memory impairment was defined as present when an individual scored three or more: the definition that has been used in all previous research to use this scale [59],[60].

#### Normal activities of daily living/instrumental activities of daily living

On the basis of responses from the CSI “D” informant interview, normal activities of daily living (ADL)/instrumental activities of daily living (IADLs) were defined as very mild or no impairment in either carrying out household chores, pursuing hobbies, using money, feeding, dressing, or toileting. The definition of impairment did not include problems arising only from physical impairments.

#### No dementia

Diagnoses of dementia were applied using the 10/66 dementia algorithm and Diagnostic and Statistical Manual of Mental Disorders IV (DSM-IV) criteria [61]. Participants meeting either criterion were excluded from the analyzed sample (both aMCI cases and controls).

### Statistical Analysis

Analyses were carried out on the 10/66 data archive release 2.1. All analyses used STATA version 10.1 [62]. As mentioned above, participants with dementia were excluded from all analyses as has been standard practice in MCI epidemiological research. Sample characteristics across countries were described including age, gender, education, number of household assets, global disability scores (WHODAS-12) [46], and NPI-Q symptoms [49].

In order to determine the potential impact of aMCI we assumed that, while both activities of daily living (ADLs) and instrumental activities of daily living (IADLs) would be expected to be intact in people with aMCI, subtle functional impairment may already be present as well as possibly nonspecific and mild behavioral and psychological symptoms of dementia (BPSD) [18]. Zero-inflated negative binomial regression (ZINB) count models were used to assess the association between aMCI and WHODAS-12 disability and NPI-Q scores using identical models to those previously reported for these samples [63]. We used zero-inflated models to deal with skewness in the distribution of the scores characterized by excessive zeros (inflation). The model distinguishes a group whose members have always zero counts (referred to as “certain zero”), from one in which members have either zero or positive counts. ZINB includes a logistic part to model the probability that a zero comes from the first group versus the second group and a negative binomial part to model the counts within the second group. Log-scale coefficients were exponentiated and 95% confidence intervals back-transformed. We determined the appropriateness of the ZINB model against a standard negative binomial model using the Vuong test postestimation and adjusted for the relevant covariates listed above, followed by Poisson regression models to generate prevalence ratios for NPI-Q symptoms as binary-dependent variables. ZINB models were further compared to zero-inflated Poisson models and in every country the test of the dispersion parameter (labelled alpha in Stata and theta by some other sources) was significant at the 0.001 level, indicating ZINB as more appropriate in all cases. Behavioural/psychological outcomes, depression, anxiety, apathy, irritability were modelled separately against aMCI as an independent variable for illustrative purposes, with no attempt to adjust given symptoms for the other three, accepting that these are related constructs.

Prevalence of aMCI was reported for each country by age and gender and adjusted for household clustering. Direct standardization, using the whole sample as the reference population, was used to compare prevalence estimates across countries after adjustment for age, gender, and education. For each country associations with age (continuous variable), gender, education (ordinal variable), and number of household assets (ordinal variable) on aMCI prevalence were calculated using mutually adjusted (as appropriate) prevalence ratios (PRs), with robust 95% confidence intervals (using the “robust” syntax in Stata to take into account household clustering: model robust standard errors [64],[65]), using Poisson working models.

To determine the pooled effects for all analyses, the statistical outputs for each country were combined into fixed-effect meta-analyses. Random effect models were not used as we wished to summarise the countries within this study rather than generalise to a hypothetical population of centres. We then calculated Cochrane Q heterogeneity and Higgins' I^2^ (95% CIs). The latter statistics set the degree of heterogeneity between studies that is not explained by chance and is expressed as a percentage with values up to 25%, 50%, and over 75% representing mild, moderate, and high heterogeneity, respectively [66].

## Results

The results were derived from a total of 15,376 participants aged 65+ and without dementia across the different countries. Response rates (i.e., participation rates for all potentially eligible residents within the defined geographic catchments) were higher than 80% in all countries. Missing data on the variables of interest were present in less than 1% of the sample. Descriptive data by country are displayed in Table 1. Age was not evenly distributed across groups (65–69, 70–74, 75–79, and 80+ y) across countries, the samples from Venezuela, China, and India being slightly younger. In all countries more women participated than men. Educational level was highest in Cuba, and the number of household assets was lowest in Mexico and India.

**Table 1:** Sociodemographic characteristics of participants by country.

Characteristics	Cuba	Dominican Republic	Peru	Venezuela	Mexico	China	India	Puerto Rico
**Sample size (** ***n*** **)**	2,620	1,767	1,767	1,820	1,821	2,014	1,802	1,765
**Response rate (%)**	94	95	82	80	85	83	83	93
**Age, ** ***n*** ** (%)** – MV	7	0	1	4	1	0	4	0
65–69 y	738 (28.2)	511 (28.9)	538 (30.5)	813 (44.7)	537 (29.5)	683 (33.9)	703 (39.0)	398 (22.6)
70–74 y	739 (28.2)	483 (27.3)	475 (26.9)	450 (24.7)	552 (30.3)	634 (31.5)	604 (33.5)	439 (24.9)
75–79 y	582 (22.2)	345 (19.5)	368 (20.8)	320 (17.6)	384 (21.1)	417 (20.7)	290 (16.1)	436 (24.7)
80+y	555 (21.2)	428 (24.2)	386 (21.8)	236 (13.0)	348 (19.1)	280 (13.9)	201 (11.2)	492 (27.9)
**Gender –** MV	0	2	0	33	0	0	15	7
Females, *n* (%)	1,686 (64.4)	1,154 (65.3)	1,073 (60.7)	1,146 (63.0)	1,143 (62.8)	1,128 (56.0)	974 (54.0)	1,183 (67.0)
**Educational level, ** ***n*** ** (%)** – MV	8	19	16	40	2	0	2	0
No education	54 (2.1)	314 (17.8)	103 (5.8)	133 (7.3)	459 (25.2)	743 (36.9)	935 (51.9)	47 (2.7)
Some education	548 (20.9)	916 (51.8)	212 (12.0)	408 (22.4)	802 (44.0)	246 (12.2)	411 (22.8)	313 (17.7)
Complete primary	864 (33.0)	338 (19.1)	654 (37.0)	913 (50.2)	337 (18.5)	532 (26.4)	301 (16.7)	356 (20.2)
Complete secondary	681 (26.0)	126 (7.1)	486 (27.5)	262 (14.4)	117 (6.4)	358 (17.8)	110 (6.1)	661 (37.5)
Complete tertiary	468 (17.9)	66 (3.7)	301 (17.0)	92 (5.1)	104 (5.7)	135 (6.7)	43 (2.4)	383 (21.7)
**Three assets or fewer** – MV	8	5	0	0	0	1	4	0
*n* (%)	67 (2.6)	256 (14.5)	83 (4.7)	33 (1.8)	373 (20.5)	104 (5.2)	918 (51.0)	4 (0.2)
**Neuropsychiatric symptoms, ** ***n*** ** (%)**	41	20	11	103	16	3	29	112
Depression	117 (4.5)	220 (12.5)	86 (4.9)	84 (4.6)	73 (4.0)	3 (0.2)	139 (7.7)	36 (2.0)
Anxiety	158 (6.0)	233 (13.2)	199 (11.3)	263 (14.5)	121 (6.6)	7 (0.4)	77 (4.3)	101 (5.7)
Apathy	117 (4.5)	226 (12.8)	93 (5.3)	138 (7.7)	165 (9.1)	15 (0.7)	18 (1.0)	58 (3.5)
Irritability	583 (22.5)	412 (23.3)	381 (21.6)	383 (21.3)	434 (23.9)	26 (1.3)	227 (12.6)	254 (15.2)
**WHODAS-12** – MV	11	15	12	96	3	12	4	9
Mean (SD)	9.69 (14.2)	13.91 (17.3)	9.36 (14.3)	9.18 (13.8)	8.59 (15.3)	5.30 (12.0)	17.44 (17.2)	12.13 (16.6)
Mean (SD) omitting zeros	16.55 (15.2)	21.11 (17.3)	15.91 (15.7)	16.18 (14.8)	18.03 (17.9)	18.39 (16.1)	22.19 (16.4)	21.33 (17.0)

MV, missing values; NPI-Q severity: total severity in neuro-psychiatric inventory.

In each country there was a statistically significant zero-inflation in the distributions of WHODAS-12 scores (Vuong test for the whole sample, *z* = 45.29, *p<*0.001) that confirmed the better fit of ZINB over negative binomial alone. Associations between aMCI, disability, and neuropsychiatric symptoms are summarized in Table 2 along with meta-analytical fixed-effect method-pooled estimates, and between-country heterogeneity. After adjustment, disability was significantly higher in aMCI cases compared to the remainder in Peru, India, and Dominican Republic, although was lower in China. The pooled fixed-effect model meta-analytical estimate indicated a positive association with disability although there was moderate to high heterogeneity in these associations between countries. After adjustment aMCI cases were more likely to have informant-rated anxiety, irritability, and apathy symptoms, with no significant between-country heterogeneity. However, there was no overall association with informant-rated depression in pooled estimates although the individual prevalence ratio was significant in Peru.

**Table 2:** Association between aMCI and disability (WHODAS-12), and the association between aMCI and neuropsychiatric symptoms (NPI–Q; depression, anxiety, apathy, and irritability).

Analysis	ZINB (95% CI)	Adjusted^a^ PRs (95% CI)			
	WHODAS-12^a^	Depression^b^	Anxiety	Apathy	Irritability^b^
**Individual study site estimates**					
Cuba	0.93 (0.74–1.19)	0.96 (0.23–3.93)	1.74 (0.77–3.94)	1.66 (0.59–4.67)	0.84 (0.44–1.57)
Dominican Republic	1.49 (1.08–2.06)	1.04 (0.47–2.30)	1.75 (1.00–3.05)	1.54 (0.76–3.12)	0.98 (0.52–1.82)
Peru	1.51 (1.17–1.94)	2.14 (1.01–4.54)	1.54 (0.89–2.65)	1.38 (0.57–3.33)	1.28 (0.83–1.96)
Venezuela	0.92 (0.53–1.60)	2.14 (0.47–9.74)	2.49 (1.40–4.42)	3.59 (1.94–6.65)	1.74 (1.06–2.86)
Mexico	1.12 (0.78–1.62)	1.07 (0.35–3.29)	1.59 (0.76–3.31)	0.79 (0.35–1.82)	1.11 (0.73–1.69)
China^c^	0.67 (0.45–0.99)	NC	NC	10.2 (1.40–74.5)	9.90 (2.57–38.0)
India	1.20 (1.03–1.40)	0.69 (0.31–1.53)	0.81 (0.25–2.57)	1.18 (0.13–10.8)	1.27 (0.82–1.98)
Puerto Rico	1.05 (0.87–1.27)	2.60 (0.90–7.54)	1.85 (0.98–3.49)	1.68 (0.65–4.34)	1.04 (0.61–1.76)
**Pooled meta-analysis (fixed-effect method)** d					
Combined estimate	1.13 (1.04–1.23)	1.31 (0.91–1.89)	1.75 (1.37–2.25)	1.83 (1.33–2.51)	1.24 (1.03–1.49)
Test for heterogeneity *p*-value	0.008	0.344	0.753	0.091	0.058
I^2^ Higgins (95% CI)	63% (20–83)	11% (0–74)	0% (0–71)	43% (0–75)	49% (0–77)

**Association between aMCI and disability** is measured by exponentiated coefficients from a zero inflated binomial model and representing the increase in disability of aMCI participants compared to normal. Zero inflation fitted using age, gender, educational level, number of household assets, depression, arthritis, visual problems, hearing problems, cough and breathing problems, heart problems, gastrointestinal problems, fainting, limb and skin problems, hypertension and stroke. **The association between aMCI and neuropsychiatric symptomsis** measured by the risk ratio from a regression using a Poisson working model and model robust standard errors, and representing the risk for having the symptom in aMCI participants compared to normal.

a Adjusted for age, gender, and educational level, number of household assets and of physical limiting impairments, psychosis, and stroke.

b Depression and irritability were additionally adjusted for pain. The four NPI–Q symptoms are all associated but in the four models presented in the table we have not adjusted each of them for the other three.

c China was not adjusted for psychosis

d The pooled fixed-effect model meta-analytical estimate for depression and anxiety were done without China.

NC, not calculable due to zero cell sizes.

The prevalence of aMCI ranged from 0.8% in China to 4.3% in India, and changed very little after direct standardization for age, gender, and education level, as displayed in Table 3. Adjusted PRs (95% CI) from Poisson regression models for independent associations with age, gender, education, and assets are shown in Table 4. No pooled associations were found with age or education but there was a modest association with male gender and fewer assets. Overall little heterogeneity was found between nations in these associations.

**Table 3:** Prevalence of aMCI by country, gender, and age group.

Country and Gender	aMCI Prevalence, % (95% CI)				Crude Prevalence (95% CI)	Standardized Prevalence (95%CI)^a^
	65–69 y	70–74 y	75–80 y	80+y	All Age Groups	All Age Groups
**Cuba (** ***n*** **)**	738	739	582	555	1.8 (1.3–2.3)	1.5 (1.0–1.9)
Males	1.5 (0.0–3.0)	1.8 (0.2–3.4)	0.0 (0.0–0.0)	1.7 (−0.2 to 3.6)	—	—
Females	2.7 (1.3–4.2)	2.6 (1.1–4.0)	1.6 (0.3–2.9)	0.8 (−0.1 to 1.7)	—	—
**Dominican Rep. (** ***n*** **)**	511	483	345	428	1.4 (0.9–2.0)	1.3 (0.7–1.8)
Males	1.7 (−0.2 to 3.6)	2.2 (0.0–4.4)	2.7 (−0.4 to 5.7)	2.9 (0.1–5.7)	—	—
Females	0.9 (−0.1 to 1.9)	1.7 (0.2–3.1)	0.4 (−0.4 to 1.3)	0.7 (−0.3 to 1.7)	—	—
**Peru (** ***n*** **)**	538	475	368	386	3.1 (2.3–3.9)	2.6 (1.9–3.3)
Males	5.4 (2.1–8.6)	2.7 (0.3–5.1)	2.1 (−0.3 to 4.5)	4.4 (1.4–7.4)	—	—
Females	2.3 (0.7–3.8)	1.7 (0.2–3.2)	3.6 (1.1–6.0)	3.4 (0.9–5.9)	—	—
**Venezuela (** ***n*** **)**	813	450	320	236	1.2 (0.7–1.7)	1.0 (0.7–1.4)
Males	1.3 (0.0–2.6)	0.0 (0.0–0.0)	2.6 (−0.3 to 5.5)	0.0 (0.0–0.0)	—	—
Females	1.6 (0.5–2.7)	1.4 (0.0–2.9)	1.5 (−0.2 to 3.1)	0.0 (0.0–0.0)	—	—
**Mexico (** ***n*** **)**	537	552	384	348	3.2 (2.4–4.1)	2.8 (2.0–3.6)
Males	3.7 (0.8–6.7)	4.3 (1.5–7.0)	5.1 (1.6–8.6)	4.0 (0.8–7.2)	—	—
Females	1.3 (0.2–2.5)	4.1 (2.0–6.2)	3.9 (1.4–6.5)	1.0 (−0.4 to 2.4)	—	—
**China (** ***n*** **)**	683	634	417	280	0.8 (0.4–1.2)	0.6 (0.3–0.9)
Males	1.0 (−0.1 to 2.1)	0.4 (−0.3 to 1.1)	1.7 (−0.2 to 3.6)	0.0 (0.0–0.0)	—	—
Females	1.3 (0.2–2.4)	0.6 (−0.2 to 1.4)	0.8 (−0.3 to 2.0)	0.7 (−0.6 to 2.0)	—	—
**India (** ***n*** **)**	703	604	290	201	4.3 (3.3–5.2)	4.6 (3.7–5.4)
Males	7.0 (4.1–9.9)	3.8 (1.5–6.1)	4.8 (1.3–8.3)	1.0 (−1.0 to 2.9)	—	—
Females	3.3 (1.5–5.0)	4.4 (2.2–6.6)	5.6 (1.8–9.5)	1.1 (−1.1 to 3.2)	—	—
**Puerto Rico (** ***n*** **)**	398	439	436	492	3.9 (3.0–4.8)	3.0 (2.2–3.8)
Males	3.9 (0.1–7.8)	5.5 (1.7–9.2)	4.1 (0.8–7.3)	5.5 (2.2–8.9)	—	—
Females	4.4 (2.1–6.8)	3.4 (1.3–5.5)	3.5 (1.3–5.6)	2.3 (0.6–3.9)	—	—

a Direct standardization for age gender and educational level using the whole sample as the standard population.

**Table 4:** Mutually adjusted (95% CI) for the independent effects of age, gender, education, and assets on aMCI prevalence.

Analysis	Adjusted PRs (95% CI)^a^			
	Age	Gender	Education	Assets
	(Per Year Increment)	(Males Versus Females)	(More Versus Less Years)	(More Versus Less)
**Individual study site estimates**				
Cuba	0.97 (0.92–1.02)	0.63 (0.33–1.21)	0.95 (0.72–1.24)	1.52 (1.00–2.30)
Dominican Republic	1.03 (0.97–1.09)	2.25 (1.04–4.86)	1.27 (0.83–1.96)	0.82 (0.63–1.06)
Peru	1.03 (0.99–1.07)	1.29 (0.75–2.22)	1.08 (0.82–1.42)	0.81 (0.64–1.03)
Venezuela	0.95 (0.88–1.02)	0.79 (0.33–1.90)	0.91 (0.55–1.52)	0.97 (0.83–1.14)
Mexico	1.01 (0.97–1.04)	1.57 (0.94–2.60)	1.24 (0.95–1.61)	0.81 (0.69–0.95)
China	0.97 (0.88–1.06)	1.00 (0.40–2.51)	0.86 (0.64–1.15)	0.80 (0.50–1.27)
India	0.97 (0.94–1.01)	1.19 (0.74–1.93)	1.14 (0.89–1.47)	0.85 (0.72–0.99)
Puerto Rico	0.99 (0.95–1.02)	1.46 (0.91–2.33)	1.04 (0.86–1.26)	0.94 (0.70–1.27)
**Pooled meta-analysis** **(fixed-effect method)**				
Combined estimate	0.99 (0.98–1.01)	1.25 (1.01–1.54)	1.06 (0.96–1.16)	0.88 (0.82–0.95)
Test for heterogeneity	0.209	0.25	0.619	0.168
Higgins (95% CI)	27% (0–67)	23% (0–64)	0% (0–68)	33% (0–70)

a Mutually adjusted for age, educational level, gender, and number of assets as appropriate.

## Discussion

Using data from a large series of cross-sectional surveys applying standard sampling and measurements, we estimated the community prevalence of Mayo Clinic–defined aMCI in six countries in Latin America, China, and India. To our knowledge this is the first study to attempt to make direct comparisons of prevalence estimates of aMCI across diverse cultures and world regions. Differences in prevalence between countries were marked and ranged from 0.8% (China) to 4.3% (India), i.e., greater than five-fold variation. After direct standardization for age, gender, and education, using the whole population as the reference, these differences were not markedly attenuated.

Inconsistencies in aMCI prevalence observed between the 10/66 study centres are likely to be due to components of the aMCI diagnosis itself. In a cross-cultural context, these support questions previously raised concerning its conceptual basis [67] and/or operationalization outside clinical settings [68]. However, aMCI has been reported to be associated with increased mortality in a prospective study [12], and differences in aMCI-associated survival between country sites cannot be excluded as a factor influencing variation in prevalence. It should be noted that the 10/66 dementia diagnosis showed much higher sensitivity than the Diagnostic and Statistical Manual of Mental Disorders IV (DSM-IV) criteria in both pilot and clinical validation 10/66 studies [41],[61]. Compared to numerous aMCI prevalence reports from community-based sites in Finland (5.3%) [26], Italy (4.9%) [69], Japan (4.9%) [32], the US (6%) [30], South Korea (9.7%) [39], Malaysia (15.4%) [38], and India (6%) [37], both the crude and adjusted aMCI prevalence reported here are relatively low. However, the estimates are similar to those reported by the British MRC CFAS study (2.5%) [15] and to estimates for aMCI prevalence in community samples from Southern France (3.2%) [33], the US (3.8%) [25], and Germany (3.1%) [70]. Low aMCI prevalence in our Latin American sites contrast with the aMCI prevalence (ranging between 3.8% and 6.3% depending on age) reported amongst American Caribbean Hispanics [31]. Differential mortality may explain these differences, but a potential role of the environment and lifestyle in the increased risk of MCI amongst Hispanic immigrants in North America cannot be excluded. Crude aMCI prevalence in India (4.3%) is similar to the figure described by Das and colleagues in Kolkata [37]. Prevalence in China was the lowest (0.6%), similar only to that described in the VITA study in Vienna [27] and markedly lower than that reported in a recent study from Chongqing (4.5%) [29]. Overall, the results suggest that there is very little consistency in prevalence of aMCI across world regions. When considered between studies, this may well reflect diagnostic issues arising from a lack of specific criteria for the operationalization of MCI (i.e., cognitive batteries and specific cut-off scores for impairment) as well as unmeasured differences and cultural variations potentially relevant for some components of the aMCI construct (such as subjective memory impairment, as described below). The objective for the analyses here was to standardize the assessments as much as possible in order to gain a clearer idea of international variation. The fact that substantial heterogeneity remains suggests important variation in constructs underlying the definition. These will be considered further below.

Female gender, increased age, lower education, and lower socioeconomic status are associated with dementia [71] and have been described in association with MCI [31]. In our study, however, the effects of age and education on aMCI prevalence were negligible across study sites, with no between-country heterogeneity in this respect. It is important to bear in mind that age- and education-standardised normative data were used to define aMCI and the lack of association supports the robustness of the norms, although for education, it might also reflect lower variance in the exposure or weaker underlying associations between education and other risk factor profiles in these samples. Lower socioeconomic status remained associated with aMCI and this may be an additional marker, beyond education, of relevant social disadvantage. The observed association with male gender contrasts with the higher reported age-adjusted prevalence of dementia in women compared to men [71], but could reflect the effect of dementia case exclusion consistent with Mayo Clinic Study of Aging reports that women experience a transition from normal cognition directly to dementia at a later age but more abruptly [20].

As described earlier, a key consideration with aMCI applied as a construct in international research is its cross-cultural validity. An advantage of the 10/66 study was that identical measures were taken and identical algorithms applied for diagnosis across the study sites and the protocols for cognitive assessments in the 10/66 study were the result of a long and painstaking process of development and validation [41]. However, a construct such as subjective memory impairment is potentially subject to cultural influences and may underlie between-site variation. For example, between sites, people with objectively lower performance on cognitive assessments may be more or less likely to admit to memory difficulties. Since this is a component of the most commonly used definitions of aMCI/MCI, these cultural variations may be reflected in differing prevalences. However, despite the differences in prevalences of aMCI between sites, associations with disability were relatively consistent, providing support for the cross-cultural applicability of the aMCI construct. They did not suggest, for example, that only more severe forms of aMCI were being identified in China where prevalence was lowest, compared to India where it was highest (particularly since disability was lower rather than higher in China in those with aMCI compared to the remainder of the sample). Associations between aMCI and disability should be viewed with caution since activities of daily living impairment is an exclusion criterion for the former. Lower likelihood of reporting difficulties in China would be unlikely to account for the negative association observed between aMCI and disability in that site because under-reporting would have to be differential between those with/without aMCI. There is very limited evidence from population-based studies on the occurrence and characteristics of neuropsychiatric symptoms that may accompany MCI [18]. While we did not find any association between aMCI and depressive symptoms, our findings of a significant association between aMCI and anxiety, apathy, and irritability are largely consistent with those from the Cardiovascular Health Study and the Mayo Clinic longitudinal study on aging in the US [72],[73], the Kungsholmen study in Sweden [74], and a small study from Thailand [75]. However, it should be borne in mind that individual behavioural/psychological symptoms were not mutually adjusted as outcomes and the independence of observed associations in Table 2 cannot be assumed.

Strengths of the study include the very large sample size and the wide range of populations sampled in terms of culture, economy, and population characteristics. Moreover, internal validity was maintained through rigorously prevalidated and standardised measurements applied consistently between countries in addition to common algorithms used to define aMCI. There are some limitations. The samples were drawn from specific geographic catchment areas and cannot be assumed to be representative of the source nation/site. No attempt was made to differentiate urban and rural status in this analysis because not all sites recruited from both settings. The study was cross-sectional in design and the impact of survival cannot be evaluated. Furthermore, within the aMCI category, participants who had developed this late in life could not be distinguished from those for whom it was a stable lifetime trait. Finally, aMCI diagnosis was determined without clinical judgement, which is difficult to obtain in large population-based studies and unfeasible in most of our study sites. Although aMCI was originally derived as a diagnosis for secondary or tertiary care clinical settings, it is being increasingly applied in epidemiological research and data from community samples is an important supplement, particularly if future community-level interventions are planned to prevent progression to dementia. Our analysis here is intended to extend this particular evidence base. Follow-up is currently underway in most 10/66 sites, which will provide further data on predictive validity.

This is one of the first studies, to our knowledge, to investigate the prevalence of aMCI in LAMICs, where the large majority of older people and people with dementia currently live [3],[4]. Longitudinal data are needed to clarify further the predictive validity of the aMCI case-definition applied here and to evaluate the extent to which it can be applied as a risk marker for further cognitive decline or dementia. In addition, further evaluation is needed of the associations with disability and neuropsychiatric symptoms since our findings do suggest higher than expected comorbidity and there are large absolute numbers of older people with aMCI in these rapidly ageing and populous world regions.

## Abbreviations

aMCI: amnestic mild cognitive impairment
CERAD: Consortium to Establish a Registry for Alzheimer's Disease
CSI “D”: Community Screening Instrument for Dementia
GMS: Geriatric Mental State
LAMIC: low- and middle-income country
MCI: mild cognitive impairment
NPI-Q: neuropsychiatric inventory
PR: prevalence ratio
SD: standard deviation
WHODAS-12: 12-item World Health Organization disability assessment schedule
ZINB: zero-inflated negative binomial regression
